# Clinical significance of different virus load of human bocavirus in patients with lower respiratory tract infection

**DOI:** 10.1038/srep20246

**Published:** 2016-02-01

**Authors:** Wujun Jiang, Fang Yin, Weifang Zhou, Yongdong Yan, Wei Ji

**Affiliations:** 1 Department of Respiratory Medicine, Children’s Hospital of Soochow University, Soochow University, Suzhou, China; 2 Department of Pediatrics, People’s Hospital of Weifang, Weifang, China; 3 Department of Infectious Disease, Children’s Hospital of Soochow University, Soochow University, Suzhou, China

## Abstract

To assess the impact of human bocavirus (HBoV) virus load on epidemiologic and clinical characteristics in children with lower respiratory tract infection (LRTI). Clinical records of a total of 654 patients with HBoV infection during January 2013 and December 2014 were retrospectively reviewed. Patients with high HBoV virus load infection had a similar age distribution with the total HBoV infection, which had a peak age group of 6–24 months. Patients with high virus load are significantly younger (P < 0.01) than those with low load. The patients who had wheeze and tachypnea/dyspnea at presentation were more strongly affiliated with the patients with high virus load (both P < 0.01). Co-infection was found significantly more frequently among patients with low virus load than those with high virus load (57.0% vs 38.9%; P < 0.01). High virus load was a significant predictor of severe LRTI (P < 0.05). HBoV infections are found in an important proportion of the hospitalized children with respiratory illnesses (8.85% in our series). A high HBoV virus load could be an etiologic agent for LRTI, which may lead to more severe lower respiratory tract symptom and severe disease.

The discovery of human bocavirus (HBoV) was the result of a viral study of respiratory secretions from Swedish children with symptoms of acute respiratory infection (ARI) reported in 2005^1^. Increasing evidences are emerging to support its role as an etiologic pathogen in lower respiratory tract infection (LRTI). Its epidemiologic and clinical characteristics have also been assessed^2^,^3^,^4^,^5^,^6^,^7^. These studies reported a prevalence of HBoV infection of 1.5%–21.5% and a coinfection rate with other viruses from 33% to 67% of specimens. The most frequent clinical diagnoses associated with respiratory HBoV infection are upper respiratory tract infections, bronchiolitis, pneumonia, bronchitis and asthma exacerbation^8^. Nevertheless, few data are available related to HBoV virus load and clinical features for children with HBoV-positive LRTIs.

In this study we aimed to assess the relationship between the HBoV virus load in respiratory tract and clinical characteristics. Samples were referred from January 2013 and December 2014, in Suzhou, China.

## Results

### Quantitative analysis of HBoV DNA in nasopharyngeal aspirates

**Figure 1:**
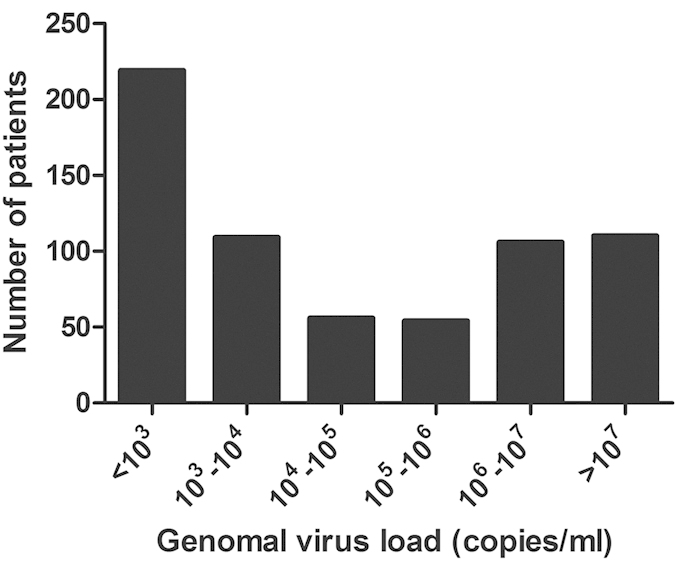
Distribution of different virus load in patients with human bocavirus infection.

Of the 7393 patients with LRTI, HBoV were positive in 654 patients with a positive rate of 8.85%. The virus load ranged from <10^3^ to 3.97 × 10^9^ copies/ml with a median of 1.2 × 10^3^copies/ml. According to the distribution of genomal virus load (Fig. 1), we classified all patients into high virus load (>10^4^ copies/ml) group (n = 326) and low virus load (≤10^4^ copies/ml) group (n = 328).

### Demographic characteristics

**Figure 2:**
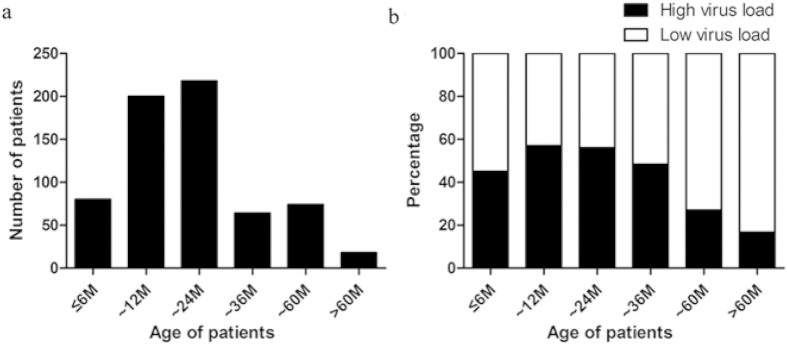
(**a**) The age distribution of the patients with human bocavirus (HBoV) infection. (**b**) Different virus load individuals categorized according to age group.

Of the 654 patients with HBoV infection, 431 (65.90%) were males and 223 (34.10%) were females. The male to female ratio was 1.93:1. The median age was 16 months (range from 1 month to 14 years). The age distribution of the patients is shown in Fig. 2A, 418 (63.91%) of the patients were aged 6-24 months.

There were no significant differences between high and low virus load in different age groups less than 36 months (p > 0.05, Fig. 2B). However, the virus load decreased with their age, with a statistical significance for age distribution (p < 0.001) in patients older than 36 months (Fig. 2B).

### Seasonal distribution of HBoV infections

**Figure 3:**
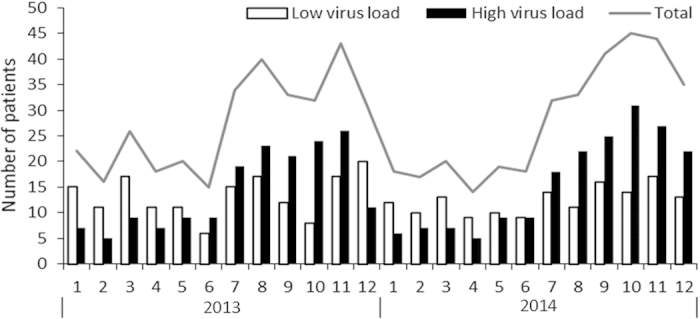
Monthly occurrence of total episodes of human bocavirus infection and cases with different virus load from 2013 to 2014.

The monthly distribution of HBoV infection is shown in Fig. 3. It occurred throughout the year with a higher proportion from July to December and it peaked in October or November. The frequency of patients with high virus load also increased from July to December and peaked in October and November. Interestingly, the frequency of patients with high virus load was higher from July to December, but lower from January to June.

### Comparison of clinical characteristics and laboratory values by PCR status

Clinical characteristics and laboratory values of children infected by HBoV with high virus load and low virus load are shown in Table 1. To exclude the interaction between parameters, mean age, fever, wheezing and tachypnea/dyspnea at presentation were tested by logistic regression analysis. On clinical manifestations, patients with high virus load were significantly younger (P < 0.01) than those with low load. The frequency of fever was significantly lower in patients with high virus load compared with low load (P < 0.01). The patients who had wheeze and tachypnea/dyspnea at presentation were more strongly affiliated with the patients with high virus load (both P < 0.01).

### Comparison of co-infection by PCR status

Of the 654 children with HBoV infection, 321 (49.1%) patients were co-infected with other respiratory pathogens, including 236 (36.1%) patients with Mycoplasma pneumoniae (MP), 46 (7.0%) with respiratory syncytial virus (RSV), 20 (3.1%) with parainfluenza virus (PIV) 1-3, 11 (1.7%) with influenza virus (IV)-A, 8 (1.2%) with adenovirus (ADV). Among the 321 cases, 36 patients were with two other pathogens.

**Figure 4:**
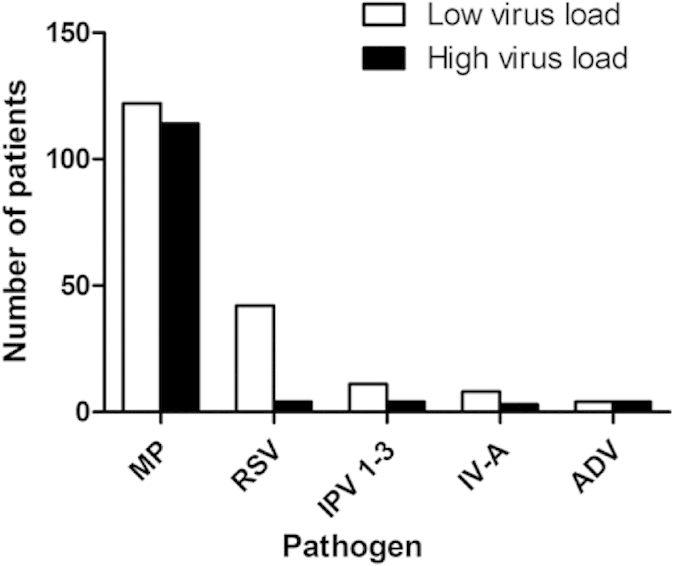
Co-infection with other pathogens in human bocavirus infection patients with different virus load. MP stands for Mycoplasma pneumoniae, RSV stands for respiratory syncytial virus, PIV stands for parainfluenza virus, IV stands for influenza virus, ADV stands for adenovirus.

Co-infection was found significantly more frequently among patients with low virus load than those with high virus load (57.0% vs 38.9%; P < 0.01). Co-infection with RSV was more strongly affiliated with the patients with low virus load (both P < 0.01, Fig. 4).

### Risk Factors for disease severity

Fifty-one (7.8%) patients received O_2_ (38 with high virus load and 13 with low virus load). None of the patients transferred to ICU. Requirement for O_2_ was used to evaluate disease severity. In unadjusted analysis, requirement for O_2_ was associated with young age, HBoV single infection and high virus load (all P < 0.05). Other variables (sex, white blood cells, percentage of neutrophils, platelets and C-reaction protein [CRP]) showed no difference.

The multivariable logistic regression model for requirement for O_2_ is shown in Table 2. Controlling for 4 demographic and clinical characteristics, significant predictors of requirement for O_2_ were age <2 months and high virus load (both P < 0.05).

## Discussion

As far as we know, this is by far the largest study focus on the clinical significance of different virus load of HBoV in patients with LRTI. We included a series of patients in the Suzhou region over two consecutive years. Also, this is the first study comprehensively describe the impact of HBoV virus load on epidemiology, clinical features, laboratory values and microbiological evaluation in children with HBoV LRTI.

During the last decade since its discovery, HBoV is found to be one of the most common pathogens that leads children to acute respiratory tract infection, especially LRTI^4^,^9^. Previous studies have shown that rates of detection of HBoV in ARI varied from 1.5% to 19.0%. In the present study, the overall detection rate of HBoV in LRTI patients was 8.85% which was similar to other regions of the world.

Age profile noted for individuals with HBoV was similar to RSV infections, with infections almost completely confine to infants and young children (<24 months). Our study are in line with most of the previous studies^1^,^2^,^10^. However, our study disagree with the Canadian study, in which there was much less difference in the prevalence of HBoV according to age^11^. Interestingly, our study also demonstrated that patients with high virus load had a similar age distribution with the total HBoV infection, which had a peak age group of 6-24 months. This interesting phenomenon highlighted the demographic importance of high virus load in HBoV LRTI.

Seasonal peaks of HBoV infection vary among different counties because of climate and geographic factors. Previous studies suggested that HBoV infection had a higher detection rate in winter^12^,^13^ or in summer^2^,^5^. In our study, a higher frequency of HBoV was observed between July and December with a peak in November. Interestingly, the frequency of patients with high virus load was also higher from July to December, and its seasonal distribution correlated with the total distribution, which highlighted the importance of high HBoV virus load in the seasonal distribution of HBoV virus.

In our study, wheezing and tachypnea occurred more frequently in children with high virus load. Actually, wheezing and tachypnea have been recognized as important clinical manifestations in HBoV infection^2^,^3^,^4^,^14^,^15^,^16^, while the clinical significance of different virus load in manifestations has been less studied. Allander etal found that high virus load of HBoV were noted mainly in the absence of other viral agents, suggesting a causative role for acute wheezing^4^. Deng etal found that wheezing was one of the most common symptoms presented by patients with positive HBoV, and the days of wheezing correlated with virus load. Different from the previous study, we use logistic regression analysis to exclude the interaction between clinical parameters and supported the fact that wheezing and tachypnea were most common and important symptoms presented in high HBoV load LRTI.

Co-infection was found significantly more frequently among patients with low virus load than those with high virus load (57.0% vs 38.9%; P < 0.01), indicating that patients with low HBoV virus load were more likely to be co-infected with other pathogens, which is consistent with the studies of Brieu^17^ and Kaida^18^. Although HBoV has been regarded as an infectious agent present, its pathogenic role in respiratory disease is still debatable. This virus is frequently detected in co-infection with other respiratory viruses of well-established pathogenic role^8^,^10^. In our study, high virus load were noted mainly in the absence of other respiratory viruses and suggesting a causative role for HBoV. However, low virus load were more detected in virus co-infection, and co-infection with RSV was more strongly affiliated with the patients with low virus load, suggesting that a low virus load in the nasopharynx seemed to be associated with long term shedding of HBoV DNA, unrelated to current illness. Actually, a recent study has suggested that asymptomatic viral infection in infants is associated with low viral load^19^.

Indeed, HBoV has been detected in infants and children with and without respiratory symptoms^19^,^20^,^21^,^22^. Byington reported that approximately half of the episodes of HBoV were asymptomatic. Furthermore, they found 14% of 151 episodes remained positive for 3 or more weeks^21^. Chonmaitree reported prolonged detection of bocavirus of up to 28 days^19^. HBoV DNA was also found in nasopharyngeal aspirates from 43% of asymptomatic children undergoing elective surgery^23^, and in lymphocytes from palatine tonsils of 32% of children undergoing tonsillectomy^24^, suggesting that HBoV could establish latent or persistent infection in respiratory tract. Actually, in our study, 18 patients readmitted within 30 days (median duration: 17 days) after discharge, while 14 (77.8%) of the readmitted patients had HBoV positive duration the second hospitalization (data not shown). This interesting phenomenon may also suggest that HBoV could establish persistent infection in respiratory tract.

Previous studies^6^,^25^ showed that the high HBoV virus load played an important role in the severity of LRTIs. Deng etal also found that high HBoV virus load led to more severe lower respiratory tract symptoms and longer hospitalization^16^. In our study, we found that age <2 months and high virus load were associated with severe LRTI (requirement for O_2_), while virus co-infection would not increase disease severity. Therefore the more severe lower respiratory tract symptom presented in high HBoV virus load patients may solely depend on HBoV virus load. Different from the previous study, our study use multivariable logistic regression which highlight the role of high virus load in severe LRTI.

The present study has potential limitations. First, quantitative PCR combined with serology would give a better idea of whether the viral infection was active or incipient^26^, we used only endpoint PCR. We found a high proportion of HBoV infections that had coinfection with other viruses. However, we may have missed additional viral infections not detected by PCR or tissue culture. As diagnostic testing for bacterial pathogens was not performed, we do not know whether HBoV-bacterial coinfections occurred among children. Because all our study subjects were hospitalized patients with LRTIs, the results are not necessarily generalizable to outpatient clinics.

In conclusion, HBoV infections are found in an important proportion of the hospitalized children with respiratory illnesses (8.85% in our series). A high HBoV virus load could be an etiologic agent for LRTI, which may lead to more severe lower respiratory tract symptom and severe disease.

## Methods

### Study Patients

All experiments were performed following the relevant guidelines and regulations of Soochow University. The methods were carried out in accordance with the approved guidelines. The study was approved by the Medical Ethics Committee of Soochow University. The parents of all study participants gave both verbal and written informed consent before study enrollment. A total of 7393 patients with a clinical and radiological diagnosis of LRTI in Children’s Hospital of Soochow University during January 2013 and December 2014 were enrolled in the study.

### Respiratory tract aspirates preparation and nucleic acid extraction

Nasopharyngeal aspirates were obtained from all patients within 24 hours of admission. This involved passing a suction catheter through the nose with the intent of passing it into the lower part of the pharynx. The depth of penetration for the nasopharyngeal aspirate catheter was set at 7–9 cm. A total of 2 ml nasopharyngeal aspirates was obtained and centrifuged at 500 × g for 10 minutes and resuspended in 2 ml saline and divided into 2 aliquots for pathogen detection using direct immunofluorescence assay (DFA) and PCR. One of the equally divided samples of nasopharyngeal aspirate was centrifuged at 12000 × g for 5 minutes, followed by extraction of DNA from a 400-ul KL sample using DNA-EZ Reagents (Sangon Biotech, Shanghai, China) or TRIzol Reagent (Life Technologies, Carlsbad, USA) in accordance with the manufacturer’s instructions. A final 200 KL of DNA was eluted and DNA sample was divided into 2 aliquots for HBoV and Mycoplasma pneumoniae (MP) gene amplification via PCR.

### Viral isolation

Direct immunofluorescence assay was done directly on nasal aspirate specimens by use of murine monoclonal antibodies (Chemicon). The specimens were tested for respiratory syncytial virus (RSV), adenovirus (ADV), parainfluenza virus (PIV) types 1–3, and influenza virus (IV) types A and B. All staining procedures were performed according to the manufacturer’s instructions. Immunostained preparations were viewed with a fluorescence microscope (Leica 020-518.500, Germany). Sputum DNA was extracted as described above, and hBoV-DNA was detected by real-time fluorescent PCR.

### Mycoplasma pneumoniae (MP) detection

Specimens were tested for the presence of MP by real-time PCR, acute IgM and IgG serology were also evaluated using enzyme-linked immunosorbent assay (ELISA). MP infection was confirmed if a positive PCR and an elevated IgM at admission or a fourfold increase in IgG at follow-up was detected.

### Data collection

Demographic, clinical features and laboratory tests including white blood cells (WBC), neutrophils, platelet, C-reaction protein (CRP) and nasopharyngeal aspirates tests were routinely performed of each patient.

### Statistical analysis

Statistical analyses were performed using the Statistical Package for the Social Sciences (SPSS; version 17.0). Data were expressed as number with percentage, mean and standard deviation (SD) or median as appropriate. Normally distributed continuous variables were compared using the Student *t* test and non-normally distributed variables were analyzed using Mann-Whitney U test. Categorical data were analyzed using the chi-squared (χ^2^) test or Fisher’s exact test. P value < 0.05 was considered statistically significant. Multivariable logistic regression analyses were conducted to identify different clinical characteristics associated with different virus load, and to evaluate independent predictors of requirement for O_2_. Factors were considered for inclusion in the model if they were found to be associated with the outcome in unadjusted analyses (P < 0.20) or were potentially clinically important.

## Additional Information

**How to cite this article**: Jiang, W. *et al.* Clinical significance of different virus load of human bocavirus in patients with lower respiratory tract infection. *Sci. Rep.* **6**, 20246; doi: 10.1038/srep20246 (2016).

## Figures and Tables

**Table 1:** Clinical characteristics and laboratory values of children infected by human bocavirus (HBoV) with high virus load and low virus load.

Parameters	high virus load (n = 326)	low virus load (n = 328)	P value
Age (m), median	16.8 ± 12.2	22.2 ± 18.4	<0.001
Clinical features			
Cough	319 (97.9)	320 (97.6)	0.80
Wheezing	118 (36.2)	41(12.5)	<0.001
Fever	169 (51.8)	199 (60.7)	0.001
Rhinorrhea	136 (41.7)	135 (41.2)	0.89
Vomiting/diarrhea	75 (23.0)	85 (25.9)	0.39
Tachypnea/dyspnea	43 (13.2)	17 (5.2)	<0.001
Median stay of hospitalization (d)	7	7	0.193
Laboratory values			
White blood cells, median (×10^9^/L)	9.6 ± 4.9	9.3 ± 4.7	0.354
Neutrophils, median (%)	47.3 ± 19.2	39.9 ± 18.2	0.024
Platelet (×10^9^/L)	370.2 ± 111.2	323 ± 114.3	0.083
C-reaction protein, median (mg/L)	1.3 ± 0.9	2.5 ± 1.1	0.113

Mean age, fever, wheezing and tachypnea/dyspnea at presentation were tested by logistic regression analysis.

**Table 2:** Multivariable predictors of requirement for O_2_ among children with human bocavirus (HBoV) infection.

Characteristics	OR (95% CI)	P value
Age in months		
<6	2.54 (1.12–5.73)	0.02
6–24	0.93 (0.74–1.11)	0.64
>24	1.00 (reference)	–
Sex		
Male	0.98 (0.82–1.49)	0.52
Female	1.00 (reference)	–
HBoV single or co-infection		
Single infection	1.39 (0.87–2.22)	0.17
Co-infection Virus load	1.00 (reference)	−
High	3.39 (1.9-6.1)	<0.01
Low	1.00 (reference)	−

Adjusted for all the variables listed in the table.
